# Endovascular management of KILT syndrome and COVID-19-related extensive deep vein thrombosis in a pregnant patient: A case report

**DOI:** 10.1016/j.radcr.2024.02.113

**Published:** 2024-03-21

**Authors:** Davide Fior, Matteo Pileri, Antonio Rovere, Lorenzo Paolo Moramarco, Domiziana Santucci, Rosario Francesco Grasso, Eliodoro Faiella

**Affiliations:** a Department of Radiology, Sant'Anna Hospital, ASST Lariana, Via Ravona 20, San Fermo della Battaglia, 22042 Como, Italy; b Operative Research Unit of Radiology and Interventional Radiology, Fondazione Policlinico Universitario Campus Bio-Medico, Via Alvaro del Portillo 200, 00128 Rome, Italy; c Research Unit of Radiology and Interventional Radiology, Department of Medicine and Surgery, Università Campus Bio-Medico di Roma, Via Alvaro del Portillo, 21, 00128 Roma, Italy; d Department of Diagnostic Radiology, San Gerardo Hospital, ASST Monza, Via Gian Battista Pergolesi 33, 20900, Monza, Italy

**Keywords:** KILT syndrome, Deep vein thrombosis (DVT), Pulmonary embolism (PE), IVC filter, SARS-CoV-2, Thrombolysis – thromboaspiration

## Abstract

We report on a 20-year-old pregnant woman who tested positive for SARS-CoV-2 and was diagnosed with KILT syndrome, a rare condition that increases the risk of thrombotic events. The patient showed signs of deep vein thrombosis that extended from the bilateral iliac vein to the inferior vena cava (IVC), which was treated with placement of an IVC filter and endovascular thromboaspiration/thrombolysis. The IVC was successfully recanalized; however, during follow-up, thrombotic restenosis occurred at the filter level, requiring filter removal. This case highlights the potential benefits of endovascular thromboaspiration/thrombolysis and IVC filter placement in patients with KILT syndrome presenting with acute thrombotic events.

## Introduction

KILT (kidney and IVC abnormalities with leg thrombosis) syndrome is a rare condition characterized by inferior vena cava and renal abnormalities that can predispose to the early development of venous thrombosis. This congenital disease should be suspected in young patients without other risk factors who experience spontaneous thrombotic events, particularly if they are bilateral [1]. Concurrent factors, such as viral infections (especially SARS-CoV-2, a well-established cause of coagulation disorders) and pregnancy, may exacerbate thrombophilia [2]. Only a few case reports on the clinical and operational management of KILT syndrome have been published in the literature thus far [3,4]. Endovascular IVC filter positioning and combined thromboaspiration/thrombolysis may present promising preventive and therapeutic options for patients with acute thrombotic events before complications such as pulmonary embolism occur.

We present a case of KILT syndrome in a patient who experienced extensive deep venous thrombosis and underwent combined endovascular thromboaspiration/thrombolysis and IVC filter placement while testing positive for SARS-CoV-2.

## Case report

A 20-year-old female patient in her 10th week of pregnancy presented to the emergency department with acute abdominal pain. Anamnesis reported congenital left renal atrophy with compensatory right renal hypertrophy and absence of previous thrombotic events. Emergency ultrasonography revealed a retained abortion and sepsis, and subsequent suction aspiration was performed to remove the embryos from the uterus.

**Fig. 1:**
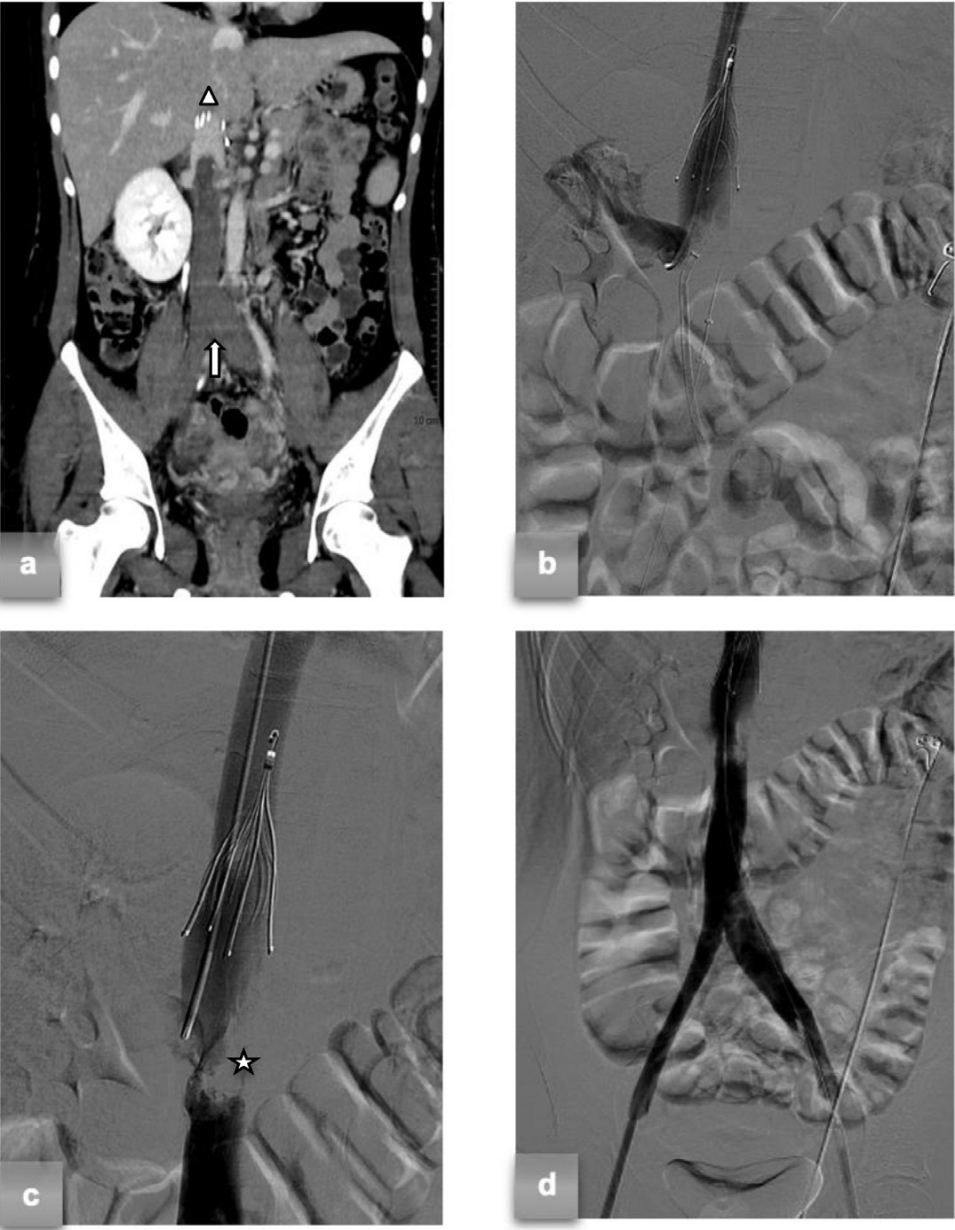
(A) Coronal CECT scan: bilateral iliac and femoral thromboses extending from IVC to the right renal vein (white arrow). Additionally, the scan showed the IVC filter positioned in the suprarenal vena cava above the thrombus level (white triangle). (B) The IVC filter was placed in the suprarenal IVC, and a catheter was inserted into the right renal vein, with the combined use of Indigo CAT-D ™ and Angiojet ™ catheters. (C) Following endovascular thromboaspiration/thrombolysis phlebographic demonstrated a focal filling defect (white star) caudal to the right renal vein with lumen stenosis of over 90%. (D) Phlebographic control after angioplasty showed a lumen patency of over 50%.

Because of persistent abdominal pain, contrast-enhanced CT (CECT) was performed, showing bilateral iliac and femoral thrombosis extending from the IVC to the right renal vein and confirming the anomaly in the left renal vein causing IVC stenosis. Contrast-enhanced chest CT scan also revealed pulmonary thromboembolic defects in the right inferior lobe. Laboratory tests revealed that the patient was heterozygous for V-Leiden factor. The molecular SARS-CoV-2 test swab, routinely performed at admission to the emergency department, was positive. The patient was later transferred to our department. The day after, in the angiography suite, a cavography confirmed the extended inferior thrombosis to the right renal vein. An IVC filter (Celect ™ Platinum - Cook) was positioned through the right jugular access in the suprarenal vena cava above the thrombus level (Fig. 1A). The patient was later transferred to a dedicated COVID-19 ward and anticoagulation therapy with heparin was initiated. Six days after her clinical condition worsened and she experienced leg swelling, a new CECT scan was conducted, revealing an extension of the thrombosis to the iliac, femoral, and popliteal axes bilaterally, as well as pulmonary thromboembolism in the right inferior lobe's segmental and subsegmental branches (Fig. 1A). Owing to resistance to anticoagulant therapy and the presence of inherent risk factors, combined endovascular recanalization therapy was selected.

From the jugular access, a 5F catheter was advanced into the right renal vein (Fig. 1B) to maintain access in case of accidental embolization during caval declotting maneuvers. Under US guidance, thrombosed common femoral veins were punctured, and 10F introducers were bilaterally positioned. A 8F x 45 cm introducer was coaxially advanced through the thrombosis from the left iliac-femoral venous axis, crossed over to the right side using a lasso catheter and guide. A combined treatment of the right iliac-femoral-popliteal venous axis was performed with a thromboaspiration catheter (Indigo CAT-D ™ - Penumbra) and a rheolytic thrombectomy with a ZelanteDVT catheter (Angiojet ™ - Boston Scientific) plus local infusion of rt-PA (Fig. 1B). Following this, the 8F x 45 cm introducer was removed, and the treatment was repeated on the left lower limb. Phlebographic control demonstrated partial recanalization of the treated venous segments, with persistence of caval thrombosis and venous discharge in the lumbar venous circles. CAT-D and ZelanteDVT catheters were advanced in parallel through both femoral accesses at the caval thrombosis level, and a second combined treatment was performed to achieve complete recanalization of the infrarenal vena cava. Following phlebographic control documented a focal filling defect caudal to the right renal vein on the medial side and a 90% reduction in the patent lumen, compatible with a nonrecent fibrotic outcome (Fig. 1C). Angioplasty with 14 mm and 18 mm balloons was performed on the stenotic segment, restoring >50% patency of the lumen (Fig. 1D). Four days after endovascular recanalization, controlled US examination confirmed the patency of the remaining deep venous districts.

The 1-month follow-up CT scan demonstrated a nonsignificant degree of thrombotic apposition in the left common iliac vein, the internal iliac veins bilaterally, and the right superficial femoral vein. Following a multidisciplinary evaluation, the patient was started on 1300 Ul/h intravenous heparin administration. The patient was discharged 14 days after treatment with heparin (subcutaneous injection of 4000 U/ twice daily) and compression bandage for the legs.

**Fig. 2:**
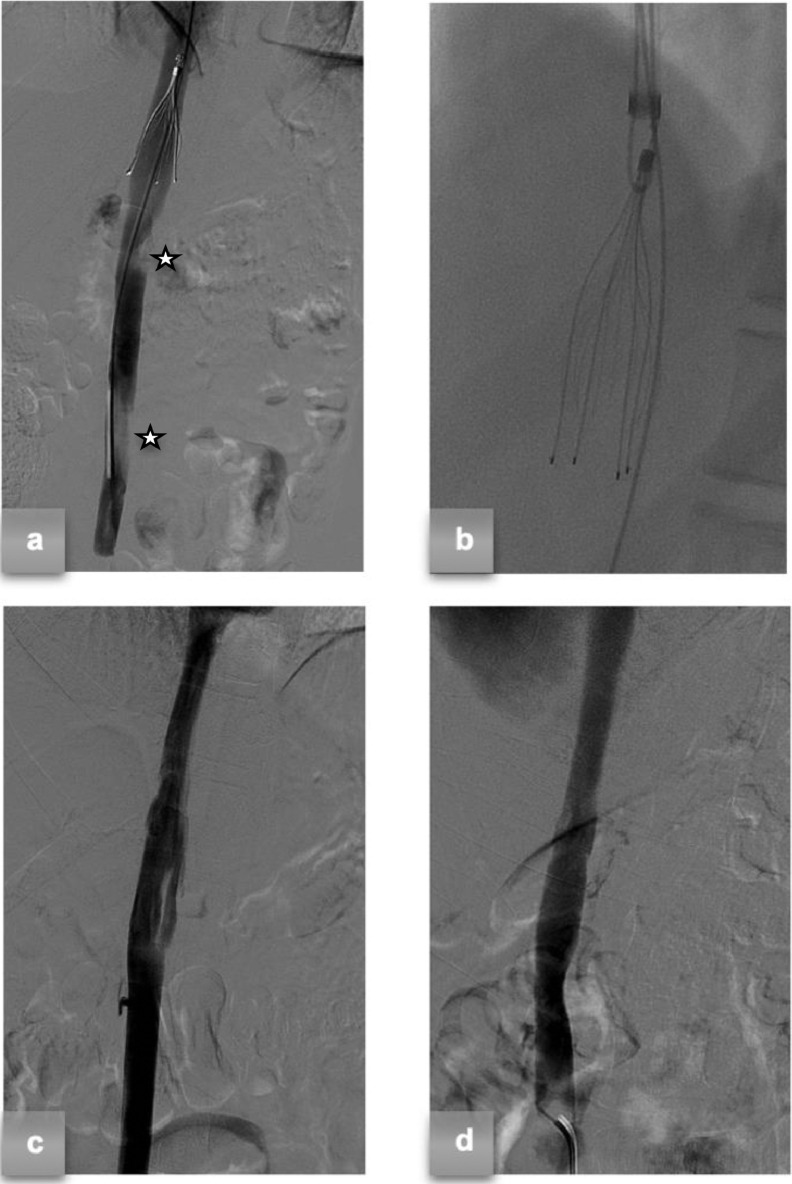
(A) Preliminar cavography confirming a severe grade of IVC restenosis (white stars). (B) Modified hangman's technique after filter realignment with a guidewire for IVC filter removal. (C) Cavography after IVC filter removal showing stenosis resolution with a concomitant tearing parietal vessel damage. (D) Final cavography after low-pressure angioplasty confirming the resolution of the stenosis.

Three weeks later, the patient returned to hospital with intense abdominal pain. Doppler US emergency examination did not show any signs of DVT; however, considering a 90% restenosis of the IVC, the decision to remove the filter and stent the stenotic segment was made. Preliminary cavography (Fig. 2A) confirmed a severe grade of restenosis under the right renal vein, embedding the filter's neck and making the retrieval unachievable from loop-snaring of the legs. After multiple attempts to break the fibrin sheath surrounding the filter using a PTA balloon (Armada™ 14 - Abbott), the hangman technique was used. This technique failed because of obstruction caused by the tilt angle of the filter and thrombotic apposition between the filter and the vein wall. Finally, successful filter removal was achieved by applying the hangman's technique after filter realignment was obtained with the aid of a right femoral guidewire through the homolateral jugular access, which operated as a cableway for the snare by being kept under tension (Fig. 2B). The procedure resulted in tearing of the IVC parietal vessel lesion (Fig. 2C), which was treated with low-pressure angioplasty (Fig. 2D). Histological examination of the fibrotic sample removed from the filter revealed fragments of venous vessels with thickening and fibrosis of the wall.

At the 4 months US follow-up examination, no residual thrombosis was identified when the patient was pregnant for 3 months.

## Discussion

This case report describes a patient diagnosed with KILT syndrome (kidney and IVC abnormalities with leg thrombosis). This rare condition can result in the early development of deep vein thrombosis, particularly in individuals with concurrent factors such as SARS-CoV-2 infection and pregnancy (10th week of gestation) [4]. The procoagulant cytokine cascade activated in COVID-19 significantly increases the risk of venous thromboembolic events and is associated with adverse outcomes [5]. The patient in this case study had several risk factors for thrombosis, including anatomical anomalies, stenosis of the IVC, left congenital renal atrophy, and being heterozygous for the V-Leiden factor, which created a challenging clinical setup for therapeutical management.

There is no clear consensus on the management of KILT syndrome. Most case reports recommend long-term anticoagulation owing to the inherent lifelong risk profile associated with IVC anomalies. Holistic multidisciplinary care, including analgesia, physical rehabilitation, and active surveillance for young-onset hypertension and renal function due to renal hypoplasia, is also recommended [6]. To prevent further episodes of pulmonary thromboembolism during the subsequent thrombectomy maneuver, an IVC filter was placed in this patient owing to the presence of numerous risk factors for thrombosis and evidence of broad thrombosis. Cavographic control revealed the presence of a thrombus in the filter, which led to the decision to keep the filter in place. In selected cases of acute DVT in the presence of inferior vena cava atresia (IVCA), endovascular approaches, such as catheter-directed thrombolysis and stenting, have been successfully applied [4,7,8]. To enhance the treatment area and minimize the risk of embolism, we applied combined therapy using a thromboaspiration catheter and a rheolytic thrombectomy catheter, which combined the advantages of both therapies (greater fragmentation capacity of the rheolytic thrombectomy catheter and greater aspiration capacity of the thromboaspiration catheter). Follow-up assessments after the initial procedure revealed non-hemodynamically significant residual thrombosis. The IVC filter removal procedure (Fig. 2B) was planned to address the stenosis that required balloon angioplasty or stenting because the filter was too proximate to the stenotic area. Although the removal process was complex, the modified Hangman technique combined with filter realignment using a guidewire was successfully implemented and should be considered as an option in challenging cases of IVC filter removal. The final cavography showed that removing the IVC filter was crucial for stenosis resolution, as it contributed to stenosis by removing part of the fibrotic tissue (Fig. 2D). This finding was crucial in deciding whether IVC stenting should be performed.

This study provides insights into the clinical and operational management of KILT syndrome. This emphasizes the importance of timely intervention to prevent complications and tailoring treatment plans to meet each patient's specific needs, particularly in cases with concurrent factors, such as viral infections and pregnancy. In many instances, conservative anticoagulant therapy is sufficient to alleviate the symptoms. As patients with KILT syndrome may be at a higher risk of thrombotic recurrence, lifelong oral anticoagulation should be considered. Additional treatments such as elastic stockings and leg elevation can also be used to prevent venous insufficiency and ulceration in these patients. Patients should be advised to avoid additional risk factors such as prolonged immobilization and oral contraceptive use [9]. Tailoring the treatment plan using an endovascular approach led to significant clinical improvement in this complex case, particularly in terms of abdominal pain, lower-extremity pain, and edema. Most importantly, the patient was able to carry her second pregnancy to full-term while receiving anticoagulant therapy.

## Conclusion

This study makes a significant contribution to the current literature on KILT syndrome by providing insightful information on diagnosing and managing this rare condition, in which interventional endovascular techniques appear to play a crucial role, resulting in improved clinical outcomes for patients.

## Patient consent

Written informed consent was obtained from the patient for publication of this case report, including accompanying images.

## Compliance with ethical standards

For this type of study formal consent is not required.

## Learning points

• Viral infections, such as SARS-CoV-2, and pregnancy can exacerbate thrombophilia in patients with KILT syndrome, leading to acute thrombotic events.
• Combined IVC filter positioning and endovascular thromboaspiration/thrombolysis can be an effective preventive option for managing KILT syndrome complications.
• The modified hangman's technique of realignment of the IVC filter with a guidewire is a successful method in complex cases of IVC filter removal.
